# Morphological and Chemical Analysis of Different Types of Calcium Silicate-Based Cements

**DOI:** 10.1155/2022/6480047

**Published:** 2022-05-19

**Authors:** Okba Mahmoud, Nashwan Abdullah Al-Afifi, Mohideen Salihu Farook, Maysara Adnan Ibrahim, Saaid Al Shehadat, Mohammed Amjed Alsaegh

**Affiliations:** ^1^Clinical Sciences Department, Faculty of Dentistry, Ajman University, Ajman, UAE; ^2^Department of Restorative Dentistry, Faculty of Dentistry, University of Malaya, Kuala Lumpur 50603, Malaysia; ^3^Department of Conservative Dentistry, Faculty of Dentistry, University of Aden, Aden, Yemen; ^4^Department of Preventive and Restorative Dentistry, College of Dental Medicine, University of Sharjah, Sharjah, UAE; ^5^Department of Oral and Craniofacial Health Sciences, College of Dental Medicine, University of Sharjah, Sharjah, UAE

## Abstract

**Objectives:**

Particle size and shape can influence the properties of materials. However, to improve the physicochemical and biological properties of mineral trioxide aggregate (MTA), silicate-based hydraulic cements were introduced. This study aimed to evaluate and compare the major constituents and crystalline structures along with the surface morphology of different types of calcium silicate-based cement (CSC).

**Materials and Methods:**

Six different types of CSC (white Portland cement, white ProRoot MTA, white MTA Angelus, Biodentine, and Endosequence, both putty and paste) were used in this study. Five samples of each material were analyzed in both uncured and cured cement using scanning electron microscopy/energy-dispersive X-ray (SEM/EDX), X-ray diffraction (XRD), and Fourier transform-infrared spectroscopy (FTIR).

**Results:**

SEM analysis showed that the surfaces of all materials consisted of particle sizes ranging from 0.194 *μ*m to approximately 51.82 *μ*m. The basic elements found in both uncured and cured cement of all tested materials using EDX were carbon, calcium, silicon, and oxygen. A difference was observed in the presence or absence of magnesium, aluminum, bismuth, zirconium, and tantalum. XRD showed that all tested materials were composed mainly of tricalcium silicate and dicalcium silicate, which are the main components of Portland cement. FTIR analysis showed aromatic rings, phosphine PH, alkyl halides, and alcohol O-H groups in all tested materials but at different wavenumbers.

**Conclusions:**

The different types of CSCs tested in this study were primarily modified types of Portland cement with the addition of radiopacifiers. ProRoot MTA and MTA Angelus contained bismuth oxide, Biodentine contains zirconium oxide, whereas Endosequence root repair materials (both putty and paste) contained zirconium oxide and tantalum oxide. Endosequence root repair materials showed smaller particle sizes than the other groups. White PC had the most irregular and large particle sizes. CSC had a smaller particle size, except for MTA Angelus. *Clinical Relevance.* The composition of CSC has a direct influence on the properties of these cements, which may affect the clinical outcome of the treatment.

## 1. Introduction

Since the early 1990s, mineral trioxide aggregate (MTA) was investigated for endodontic applications and consequently approved for use by the U.S. Food and Drug Administration in 1998 [1]. MTA was considered the first patented hydraulic calcium silicate-based cement (CSC) for endodontic use [2]. It has been used for treating a variety of root canal perforations, apexification, pulpotomy, and the treatment of the vital pulp (pulp capping) [3, 4]. A 10-year follow-up study performed by Daniele [5] revealed that MTA can be effectively used to treat the exposed pulp by direct pulp capping with a 92.5% success rate. More recently, CSCs have been used as root canal sealers in dentine hypersensitivity treatment [6, 7]. In addition, teeth with necrotic pulp and open apex can be treated with CSC to produce an apical barrier (apexification). When CSC comes into contact with synthetic tissue fluids, hydroxyapatite crystal formation begins, which results in the formation of calcified structures [3, 8]. In fact, chemical analysis of elements present in MTA and Portland cement (PC) showed that PC has chemical elements similar to MTA, except for bismuth, which is only found in MTA [9–11]. For this reason, PC has been studied extensively as an alternative to MTA [12]. Although PC can be claimed as an MTA substitute, it is important to emphasize that PC and MTA are not the same materials [11].

The MTA powder comprises fine hydrophilic particles with its basic compounds being: tricalcium silicate, dicalcium silicate, tricalcium aluminate, tricalcium oxide, and silicate oxide. In addition, bismuth oxide, which is used as a radiopaciﬁer, makes MTA visible on radiographs [13]. Recently, to improve the physicochemical and biological properties of MTA, silicate-based hydraulic cement was introduced into the market [13–15].

Biodentine is considered a relatively new CSC that was developed to transcend MTA cement and replace it in endodontic applications [16]. It is available as a capsule, containing a perfect proportion of its powder and liquid. However, the manufacturer does not provide the precise concentration of Biodentine components [17]; this is reported in a study by Camilleri et al. [18]. Some of the disadvantages of MTA, considered in Biodentine, are difficult manipulation, high cost, and slow setting [17]. Han and Okiji [19] evaluated the root canal dentine uptake from Biodentine and ProRoot MTA and found that the dentine uptake of calcium and silicon was more notable for Biodentine compared to MTA.

The endosequence root repair material (ERRM) putty and paste (Brasseler, Savannah, GA, USA) have also been developed as ready-to-use, premixed bioceramic materials, advocated for apical surgical procedures, pulp capping, perforation repair, and apical plug [20]. These materials generally have a chemical composition that is broadly similar to MTA and monobasic calcium phosphate [21].

Some materials or solutions have been incorporated into some MTA types of cement to improve its qualities. The addition of CaCl (2) improved the sealing ability of MTA cement [22]. However, some additions may negatively affect the physical and chemical properties of the original materials. For example, if a chemical additive is added to cement before it is hydrated, it will affect the hydration process and may have a negative impact on the cement itself [23]. It has been reported that particle size and surface area play an important role in the physicochemical properties of the material [24].

There are a wide variety of CSCs currently available in the market. Therefore, this study aimed to investigate and compare the surface morphology and chemical composition of the most common types of CSC using scanning electron microscopy/energy-dispersive X-ray (SEM/EDX), X-ray diffraction (XRD), and Fourier transform-infrared spectroscopy (FTIR).

## 2. Materials and Methods

Six different types of CSC used in this study are shown in Table 1.

### 2.1. Surface Morphological Analysis and Major Constituents of CSC Using Scanning Electron Microscopy/Energy Dispersive X-Ray (SEM/EDX)

#### 2.1.1. Preparation of Uncured Cement

Five samples from each material (*n* = 5) were examined using SEM. The uncured cement of the tested materials was lightly interspersed over the surface of an aluminum stub with double-sided carbon tape to provide electrical conductivity. The sample was subsequently mounted on a 7-holder sample stage inside the SEM chamber. Imaging was performed at 10 or 20 kV accelerating voltage and 9–14 mm working distance at ×500, ×1000, and ×2000 magnifications using secondary electron signals. The sizes of the cement particles were determined using the scale provided in each micrograph. EDX was used in combination with SEM to determine the constituent elements and chemical compositions of the cement. EDX analysis was performed twice for each sample.

#### 2.1.2. Preparation of Cured Cements

Each tested material (Table 1) was mixed and handled according to the manufacturer's instructions (a water/powder ratio of 1 : 3 by weight was used) by one operator. Five disc-shaped samples (*n* = 5) (5 mm diameter × 2 mm height) were fabricated for each material by packing the cement into a Perspex mold (Zecttron, Malaysia). Then, the assembly (for each specimen) was wrapped with gauze moistened with deionized water and kept inside an incubator (Memmert GmbH, Schwabach, Germany) at 37°C and 100% humidity for 3 days, until complete curing of the material prior to the examination as previously described [25].

The specimens were then mounted directly on aluminum stubs and viewed using SEM. The surfaces of the samples were examined, and the centers of the specimens were further examined after fracturing on a glass slab with a spatula. Imaging was performed under 10 or 20 kV accelerating voltage and 9–14 mm working distance at ×500, ×1000, and ×2000 magnifications using secondary electron signals. EDX chemical analysis and surface morphological examination were performed twice for each sample in the different areas.

Based on the study performed by Kung et al. [26], the diameter of dentin tubules is about 2–5 *μ*m, according to the SEM image of the dentin specimen. Therefore, in the present study, a particle size smaller than 2 *μ*m is considered small, while a particle size bigger than 2 *μ*m is considered large.

### 2.2. The Major Compounds and Crystalline Structures of CSC Using X-Ray Diffraction (XRD)

#### 2.2.1. Preparation of Uncured Cement

The uncured cement (*n* = 5) was lightly interspersed over the X-ray holder and then compacted on it by applying slight pressure using a dental spatula. Excess cement was removed from the surface of the X-ray holder using a single sweep with the edge of a glass slide. The holder was visually checked to ensure that uniform and complete coverage of the holder was achieved. The X-ray holder was placed on a metal slide and mounted onto an X-ray diffractometer (PANalytical X'Pert Pro diffractometer) before the phase analysis of each material.

#### 2.2.2. Preparation of Cured Cements

The tested materials (*n* = 5) were prepared in the same way as described previously (Section 2.1.2). The cured sample was aseptically harvested by gently scraping the crystals with a small, sterile dental mixing spatula.

For both uncured and cured cement, phase analysis was performed using XRD in an automated powder diffractometer using CuKa radiation and a secondary crystal monochromator. A D8 advanced X-ray diffractometer operated at 40 kV and 40 mA with CuKa radiation (1.5406 Amstrong) was used. The diffraction pattern of the unknown material was compared with the documented diffraction patterns of known materials to assess the phases in the tested materials. The diffraction patterns of known materials are documented in the Joint Committee of Powder Diffraction Standard International Center for Diffraction Data (JCPDS-ICDD) files.

### 2.3. Characterizations of CSC Phases Using Fourier Transform-Infrared Spectroscopy (FTIR)

#### 2.3.1. Preparation of Uncured Cement

The tested materials were analyzed using a Smart iTR Diamond ATR system (FTIR Spectrometer) with a DTGS KBr detector between 4000 and 525 cm^−1^ range at a resolution of 4 cm obtained using 32 scans.

A small amount of uncured cement (*n* = 5) was placed in a hole located on the device's window (diamond). Then, the head of the holder was manually adjusted until it came into contact with the cement material. The scan was then started, and the results from the software (OMNIC 8.1, Thermo Fisher Scientific Inc. 2009) were collected.

#### 2.3.2. Preparation of Cured Cements

Each material was prepared in the same way as described in Section 1.2. The cured sample was then crushed aseptically using a pestle and mortar. The crushed cement was placed in a hole in the window (diamond), as described previously in Section 3.1.

### 2.4. Statistical Analysis

The data were analyzed for normal distribution; then, a one-way ANOVA followed by the Tukey post hoc test was used for statistical analysis at a significance level of *P* < 0.05.

## 3. Results

### 3.1. Surface Morphological Analysis and Major Constituents of CSC Using Scanning Electron Microscopy/Energy Dispersive X-Ray (SEM/EDX)

#### 3.1.1. SEM Analysis of Uncured Cement

**Figure 1:**
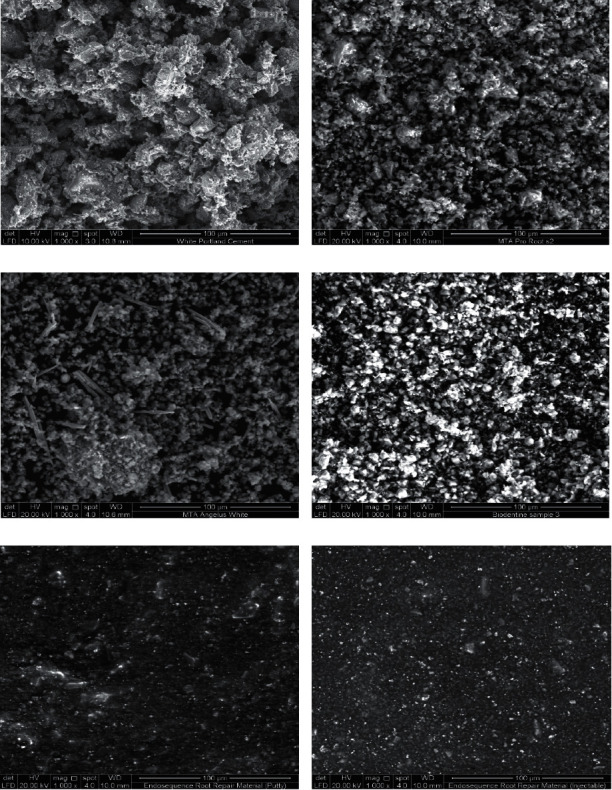
Scanning electron micrographs of uncured cement (×1000). (a) White PC. (b) White ProRoot MTA. (c) White MTA Angelus. (d) Biodentine. (e) ERRM putty. (f) ERRM paste, showing the differences in particle sizes and shapes between the different types of CSC.

The scanning electron micrographs (×1000) of uncured cement tested in this study are shown in Figure 1.

Visual examination of uncured cement by SEM showed that CSC contained crystalline particles, which were coarse and irregular in shape, scattered throughout a general groundmass of finer amorphous materials. The particle sizes and shapes differed between the tested materials (Figure 1).

The smallest particle size was found in the ERRM putty and paste (Figures 1(e) and 1(f)), while the largest particle size was found in white PC and white MTA Angelus, with the largest particles in MTA Angelus having an elongated shape (Figures 1(a) and 1(c)). The homogeneity in size and shape of the particles has been largely observed in both types of ERRM (putty and paste), followed by white ProRoot MTA and Biodentine. Less homogeneity of the particles was observed in white PC and white MTA Angelus.

Overall, the SEM results revealed that the microstructures of the CSCs were crystalline and composed of spherical, cubic, and/or needle-like crystals (Figure 1).

The mean and standard deviation of the small and large particle sizes of the uncured CSC are shown in Table 2. The particle sizes were significantly different between the groups (*P* < 0.05).

The small particle size of uncured CSC was found in the ERRM putty and paste, followed by Biodentine. However, MTA Angelus has the largest size of small particles. A post hoc Tukey test showed significant differences lie between white PC and ERRM putty and paste, as shown in Table 3. The large particle sizes of uncured CSC have been shown in MTA Angelus and white PC which are the largest size of the large particle followed by Pro-Root MTA and Biodentine, ERRM (putty and paste), respectively. A post hoc Tukey test showed significant differences between white PC and the other uncured CSC, as shown in Table 4. However, MTA Angelus has the largest size of large particles.

#### 3.1.2. SEM Analysis of Cured Cement

**Figure 2:**
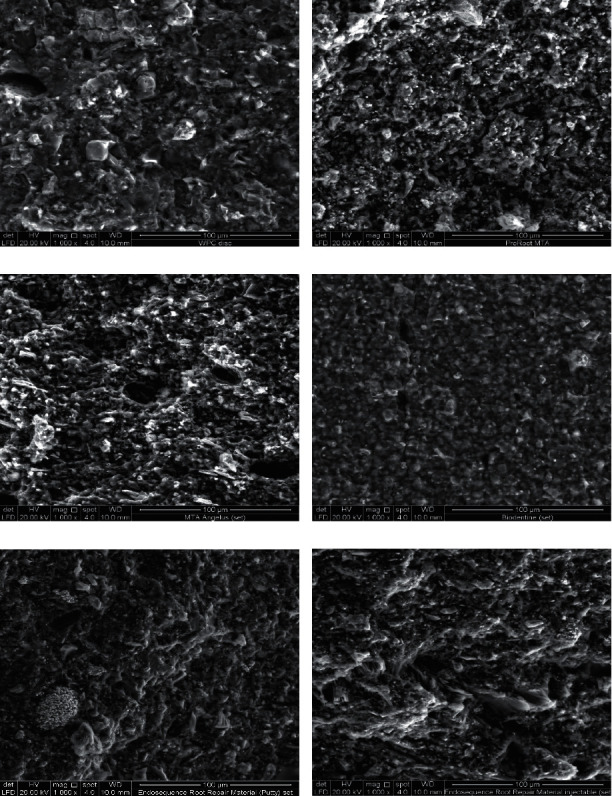
Scanning electron micrographs of cured cement (×1000). (a) White PC. (b) White ProRoot MTA. (c) White MTA Angelus. (d) Biodentine. (e) ERRM putty. (f) ERRM paste, showing the differences in particle sizes and shapes between the different types of CSC.

The scanning electron micrographs (×1000) of the cured cement tested in this study are shown in Figure 2. Visual examination of cured cement by SEM showed that the particle sizes and shapes differed between the tested materials (Figure 2).

The smallest particle size was found in the ERRM putty and paste (Figures 2(e) and 2(f), respectively), while the largest particle size was found in white PC and white MTA Angelus; however, the largest particles in MTA Angelus had an elongated shape similar to that in uncured cement (Figures 2(a) and 2(c), respectively). The homogeneity of the cement particles was largely observed in both types of ERRM (putty and paste) followed by Biodentine. Less homogeneity of the cement particles was found in the white PC. The nature of the relatively low circularity elongated and sharp/large-size population is presented in white PC.

Moreover, cement tag-like structures were observed within the cement surface of both types of ERRM (putty and paste). However, no tag-like structures were observed in the remaining cured materials.

Scanning electron micrographs demonstrated that white PC was composed of particles with larger sizes and irregular shapes, whereas the particle size and shape were more homogeneous in the dental CSC (Figure 2).

The mean and standard deviation of the small and large particle sizes of the cured CSC are listed in Table 5. The size of the particles was significantly different between the groups (*P* < 0.05).

The small particle size of cured CSC was observed in the ERRM putty and paste, followed by white PC. A post hoc Tukey test showed that the significant differences were between white PC, Pro-Root MTA, MTA Angelus, and Biodentine, as shown in Table 6. However, Biodentine had the largest size of the small particles of cured CSC. The large particle size of cured CSC has been shown in MTA Angelus and white PC followed by Pro-Root MTA, Biodentine, and ERRM (putty and paste). ERRM (putty and paste) presented the smallest size of the large particles. A post hoc Tukey test showed significant differences between white PC and the other cured CSC, as shown in Table 7. However, MTA Angelus has the largest size of the large particles of cured CSC.

#### 3.1.3. EDX Analysis of Uncured and Cured Cement

**Figure 3:**
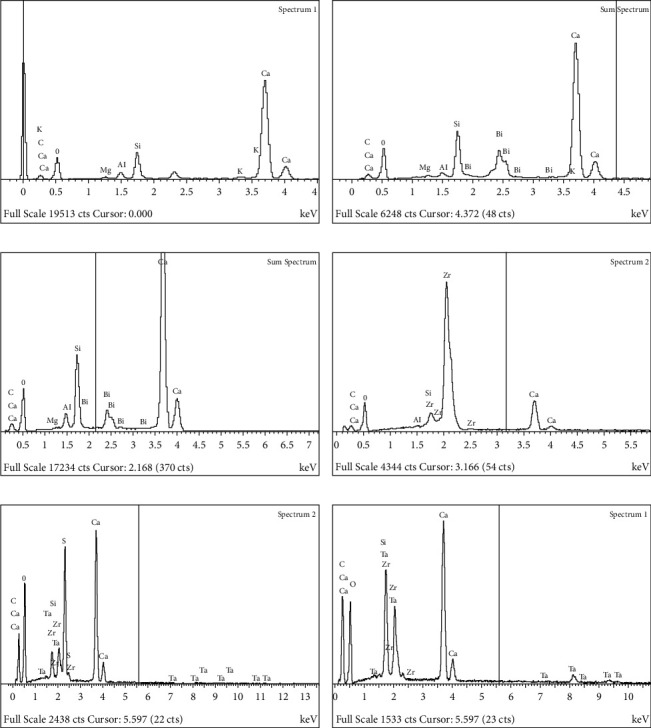
(a–f) Energy dispersive X-ray analysis (EDX) spectrum of uncured cement (X350), showing that all types of CSC were composed primarily of carbon (C), calcium (Ca), silicon (Si), and oxygen (O) whereas radiopacifier was absent in white PC. Radiopacifier presented as bismuth oxide (Bi) in MTA, zirconium oxide (Zr) in Biodentine, and both zirconium oxide (Zr) and tantalum oxide (Ta) in ERRM putty and paste.

The EDX results of the uncured cement tested in this study are shown in Figure 3. These figures show the elemental peaks for each material. The same elements are shown in both uncured and cured cement of the tested materials. The chemical compositions of uncured cement of different types of CSCs are listed in Table 8.

EDX showed that all CSCs, including PC, were composed primarily of the same elements: carbon, calcium, silicon, and oxygen. The dental CSC differed from white PC principally by the inclusion of radiopacifier (bismuth oxide in MTA, zirconium oxide in Biodentine, and both zirconium oxide and tantalum oxide in ERRM putty and paste).

Moreover, white PC, ProRoot MTA, MTA Angelus, and Biodentine contain aluminum ions which are absent in both ERRM putty and paste. Biodentine and both ERRM putty and paste differed from PC and other tested CSC due to the lack of magnesium. The white PC differed from the other CSCs by the presence of potassium and the absence of a radiopacifier.

### 3.2. Major Compounds and Crystalline Structures of CSCs Using X-Ray Diffraction (XRD)

#### 3.2.1. XRD Analysis of Uncured and Cured Cement

**Figure 4:**
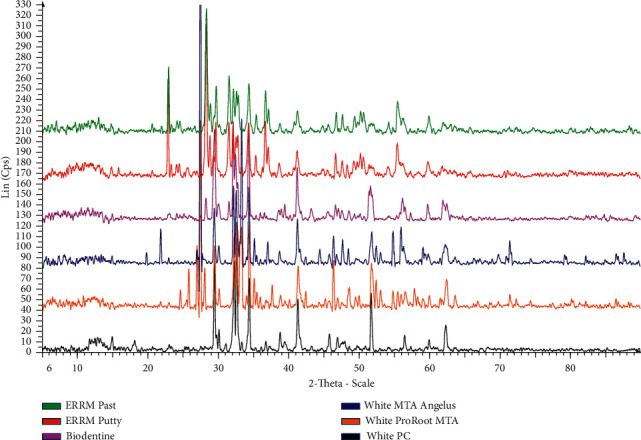
XRD patterns of different types of uncured CSC showing the main crystal phases of each cement, namely, tricalcium silicate, dicalcium silicate, and tricalcium aluminate.

**Figure 5:**
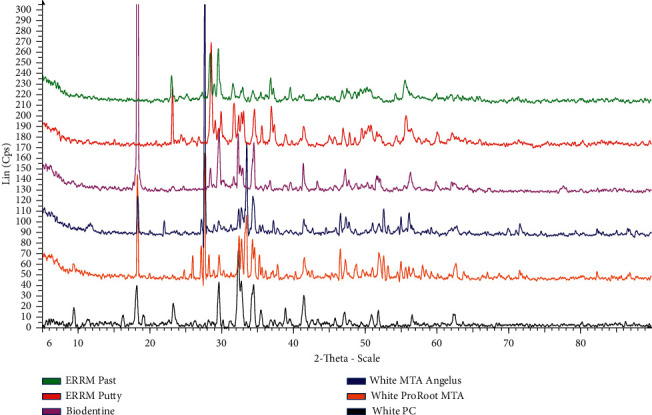
XRD patterns of different types of cured CSC showing the main crystal phases of each cement, namely, tricalcium silicate, dicalcium silicate, and tricalcium aluminate.

The major compounds and crystalline structures of different types of uncured and cured CSC are shown in Table 9 and Figures 4 and 5.

XRD analysis showed that all uncured and cured CSC tested in this study were composed mainly of tricalcium silicate (Ca_3_SiO_5_), dicalcium silicate (Ca_2_SiO_4_), and tricalcium aluminate (Ca_3_Al_2_O_6_), which are the main compounds in white PC.

Radiopacifiers were only detected in dental CSCs (uncured and cured). A bismuth oxide crystalline structure was found in white ProRoot MTA and white MTA Angelus, whereas zirconium oxide (ZrO_2_) was found in Biodentine, ERRM putty, and ERRM paste, and tantalum oxide (Ta2O5) was only found in both ERRM putty and paste. White PC did not contain bismuth oxide, zirconium oxide, or tantalum oxide. Furthermore, XRD showed that anhydrous calcium hydroxide [Ca (OH)_2_] was found in white PC, ERRM putty, and ERRM paste, while it was missing in the remaining uncured CSCs (Figure 4 and Table 9). However, calcium hydroxide [Ca (OH)_2_] was found in all cured CSCs, including white PC (Figure 5).

After hydration, the main phases of tricalcium silicate, dicalcium silicate, and tricalcium aluminate were identified at the same locations as those for uncured CSC (see Figures 4 and 5).

There was no difference in major crystal phases between the examined uncured and cured cement specimens, except for the white PC, ERRM putty, and paste containing Ca (OH)_2_ in both forms (uncured and cured). Other crystal phases contributed to only a small proportion of the materials, and many of them could not be identified. There were slight differences in the patterns of the various CSCs tested in this study.

### 3.3. Characterizations of CSC (Uncured and Cured) Phases Using Fourier Transform-Infrared Spectroscopy (FTIR)

**Figure 6:**
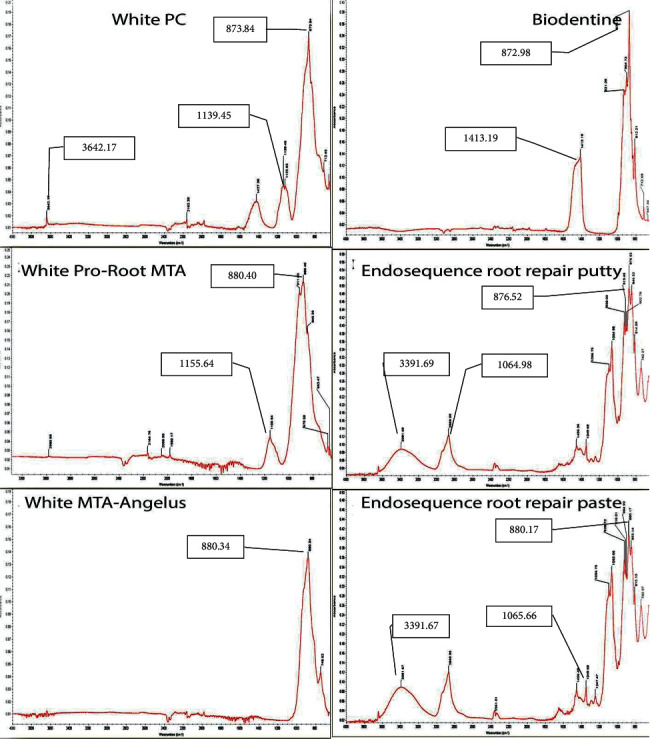
The FTIR spectrum of different types of uncured CSC showing the absorbance peaks of the precipitates of each cement. The absorbance peaks ranging from 872.98 to 3642.17 were seen.

**Figure 7:**
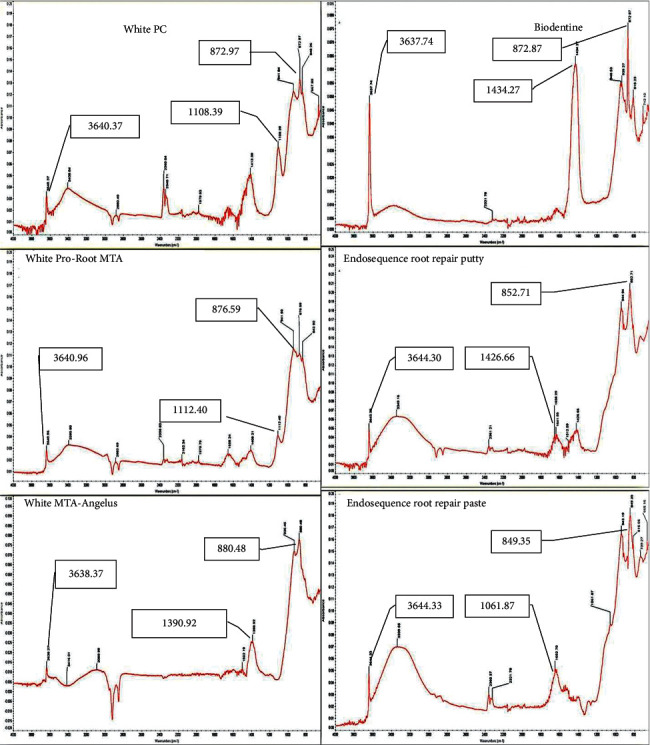
The FTIR spectrum of different types of cured CSC showing the absorbance peaks of the precipitates of each cement. The absorbance peaks ranging from 849.35 to 3644.33 were seen.

The FTIR spectra of different types of uncured and cured CSC tested in this study are presented in Table 10 and Figure 6 (uncured cement) and Table 11 and Figure 7 (cured cement). In general, the wavenumbers of FTIR have been described and classified according to the FTIR table as follows: aromatic C-H bend (meta) ∼880 cm^–1^ and C-H bend (para) 850–800 cm^–1^. Phosphines PH bend 1090–810 cm^–1^ and alkyl halides C-F stretch 1400–1000 cm^–1^. Alcohol O-H stretch ∼3650 or 3400–3300 cm^–1^ and C-O stretch at 1260–1000 cm^–1^.

FTIR analysis showed aromatic rings, phosphine PH, alkyl halides, and alcohol O-H groups were found in all tested materials but at different wavenumbers.

#### 3.3.1. FTIR of Uncured Cement

The FTIR absorbance spectra of the precipitates of different uncured CSCs are shown in Table 10 and Figure 6. The absorption peaks at 873.84, 1139.46, and 3642.17 cm^−1^ for white PC, 880.40 and 1155.64 cm^−1^ for white ProRoot MTA, 880.34 cm^−1^ for white MTA Angelus, 872.98 and 1413.19 cm^−1^ for Biodentine, 876.52, 1064.98, and 3391.69 cm^−1^ for ERRM putty, and 880.17, 1065.66, and 3391.67 cm^−1^ for the ERRM paste were identified (Table 10).

#### 3.3.2. FTIR of Cured Form

The FTIR absorbance spectra of the precipitates on different cured CSCs are shown in Table 11 and Figure 7. The absorption peaks at 872.97, 1108.39, and 3640.37 cm^−1^ for white PC, 876.59, 1112.40, and 3640.96 cm^−1^ for white ProRoot MTA, 880.48, 1390.92, and 3638.37 cm^−1^ for white MTA Angelus, 872.87, 1434.27, and 3637.74 cm^−1^ for Biodentine, 852.71, 1426.66, and 3643.30 cm^−1^ for ERRM putty, and 849.35, 1061.87, and 3644.33 cm^−1^ for ERRM paste were identified (Table 11).

## 4. Discussion

Chemical analysis and characterization of different types of CSC were performed using a combination of SEM/EDX, XRD, and FTIR analyses. SEM is considered the most frequently used technique for microstructure observation of biomaterials [27], and in combination with EDX, it provides information about the elemental composition of the test materials [18, 28]. Therefore, in the present study, SEM and EDX were used to characterize the microstructure and constituent elements of various types of CSC.

In the present study, scanning electron micrographs showed that large and irregular particles were present in white PC. This is in agreement with a study performed by Hwang et al. [29], who reported that white PC had irregular and larger particle sizes compared to white ProRoot MTA. In another study, white MTA Angelus was shown to have particles with relatively low circularity and wide size distribution and was less homogeneous in comparison to white ProRoot MTA [30]. This coincides with our findings in scanning electron micrographs. Furthermore, Song et al. [31] reported that white PC has a less homogeneous composition than white ProRoot MTA. Another study demonstrated that MTA Angelus has multiple aggregates of round particles in which long spindle-shaped particles are detected [32]. Similarly, large particles with an elongated shape were observed in MTA Angelus in this study. Regarding their size and shape, the small size of the particles penetrated the dentinal tubules more easily and more deeply, even in the presence of intracanal medicament residues on the wall of the root canal [33]. As mentioned elsewhere, regarding the size and shape of MTA particles, it has been found that small MTA particles (size, 1.5 *μ*m) made it possible to go into open dentine tubules (2–5 *μ*m) [30]. Therefore, it can be concluded that ERRM has a better ability to penetrate dentine tubules as it has the smallest particle size [30]. In addition, the debonding force to the root dentin is decreased when a cement contained a smaller particle size [33]. This may be an important mechanism for the provision of hydraulic seals. The particle size and shape distribution may play an important role in the handling characteristics of MTA, which may help increase the surface area and increase the reactivity of particles (dicalcium silicate and tricalcium silicate) to form calcium hydroxide and calcium silicate hydrate phases [30]. In addition, MTA setting time can be accelerated and regulated by reducing the particle size distribution without adding chemicals [34].

Jalloul et al. [35] evaluated the chemical and morphological structure of Biodentine and glass ionomer cement and revealed that the surface of Biodentine was spherical when immersed in deionized water, and needle-like crystals of Ca (OH)_2_ were detected when immersed in phosphate solution. In another study, the surface of the ERRM (putty) is presented with small, irregular particles which were interrupted by small, rounded globular (spherical) crystals and a few needle-like particles. The surface of ERRM (paste) is composed of small-sized particles and aggregates of medium-sized grains with scattered rod-like bundles [25]. These results agreed with those of the present study, where the SEM results demonstrated that all CSCs were composed of spherical, cubic, and/or needle-like crystals. During the hydration process, two calcium silicates react and form needle-like amorphous calcium–silicate–hydrate (C–S–H) gels and columnar-shaped calcium hydroxide crystals that intermingle with each other and consolidate, which is known to provide the primary strength of the material [36].

In this study, the scanning electron micrograph showed that both ERRM (putty and paste) produced tag-like structures within the cement itself; however, no dentin–cement interface was observed because no tooth was used in this study. Nevertheless, Abu Zeid et al. [25] demonstrated that there was a gap at the Endosequence putty-dentine interface, whereas the Endosequence paste-dentin interface was sealed with the material itself when kept in deionized water. However, both material (putty and paste) dentine interfaces were filled with spherulites and acicular crystals when placed in fetal calf serum. Interestingly, endosequence materials produced tag-like structures inside the cement itself, which were better than the other tested materials in this study. The tag-like structures could be an indicator of a high calcium ion release that contributes to high dislodgement resistance and provides better micromechanical adhesion [37]. In an in vitro study conducted to evaluate the bioactivity and physicochemical properties of various CSCs (MM-MTA, Biodentine, and ERRM putty), ERRM putty showed the highest rate of Ca^2+^ release [38], which could be related to the time required for the final setting of this material, which appeared after almost 200 min in a humid environment during the first 24 h [39]. EDX identiﬁes the constituent elements of the material [40, 41]. In a study performed by Camilleri et al. [40], white PC and white Pro-Root MTA have the same constituent elements, namely, calcium, silicon, and aluminum. In addition, a study performed by Estrela, Bammann [9], and Song et al. [31] showed that the difference between white PC and white ProRoot MTA is the absence of bismuth oxide ions and the presence of potassium ions in white PC. The two commercial brands of white MTA (ProRoot and Angelus) have a similar chemical composition, and bismuth is shown only in white MTA cement to provide radiopacity [42]. This is in agreement with the results of the present study where no difference in the constituent elements between white ProRoot MTA and white MTA Angelus was observed. Marciano, Duarte [43], and Voveraityte et al. [44] reported that bismuth oxide is the reason for tooth discoloration; that is, it changes in color from light yellow to dark brown when in contact with sodium hypochlorite residues in dentinal tubules. Another study mentioned that to reduce the effect of tooth discoloration, final irrigation with water before obturation is recommended [44]. Several methods for preventing tooth discoloration caused by MTA and MTA-like cement have been proposed, including the application of dentin bonding agents on dentinal walls, the use of cement containing radiopacifying agents other than bismuth oxide, and the addition of zinc oxide to cement containing bismuth oxide [45].

Another study using EDX for the chemical analysis of ERRM detected the following elements: calcium (Ca), oxygen (O), carbon (C), phosphorus (P), zirconium (Zr), tantalum (Ta), silicon (Si), and sodium (Na), with traces of fluoride (F), magnesium (Mg), aluminum (Al), and sulfur (S) [25]. In the present study, some elements such as phosphorus and sodium, in addition to the trace elements of fluoride, magnesium, aluminum, and sulfur, were not detected in either type of ERRM (putty and paste). Magnesium ions were present in the remaining tested materials, except Biodentine. This is in agreement with a previously reported study, where EDX analysis revealed that Biodentine is composed of carbon, oxygen, aluminum, silicon, calcium, and zirconium [46].

Among the various trace elements, magnesium is a mineral element that has a stimulatory influence on the development of bone. The results of biological properties such as cytotoxicity improved with magnesium ions of calcium silicate cement (CSC) [47].

The EDX results of the present study showed that the chemical compositions of both types of ERRM (putty and paste) were similar and contained calcium, silicon, oxygen, zirconium, and tantalum.

XRD determines the phases present in uncured and cured specimens [48]. XRD was used to investigate the phase composition of the specimens. In the present study, the chemical composition and crystalline structure of white PC and CSCs using XRD were similar. A difference was observed in the presence of bismuth oxide (Bi_2_O_3_) in white MTA (ProRoot and Angelus), in the presence of zirconium oxide (ZrO_2_) in Biodentine and both types of ERRM (putty and paste) materials, and in the presence of Tantite (Ta_2_O_5_) in both types of ERRM (putty and paste) materials. This result was in line with the study performed by Camilleri et al. [40], who reported that white PC and white ProRoot MTA are composed primarily of tricalcium and dicalcium silicate. In addition, Song et al. [31] showed that white PC and white ProRoot MTA were composed mainly of bismuth oxide crystalline structure and calcium silicate oxide. Moreover, a study performed by Basturk et al. [49] demonstrated that both materials (Pro-Root MTA and MTA Angelus) are composed of tricalcium silicate (Ca_3_SiO_5_), dicalcium silicate (Ca_2_SiO_4_), bismuth oxide (Bi_2_O_3_), and calcium hydroxide (Ca (OH)_2_), as presented in this study, in addition to calcium carbonate (CaCO_3_), which was not detected in this study. XRD analysis in this study showed that the chemical compositions were similar in both endosequence root repair material types (putty and paste). This is partially in agreement with the manufacturer of Endosequence root repair materials (Brasseler USA, Savannah, GA) [50], who mentioned the same composition for both types, except for the presence of calcium sulfate instead of calcium aluminum oxide (Ca_3_Al_2_O_6_) presented in this study. Both materials contained tantalum oxide [Tantite, Ta_2_O_5_] and zirconium oxide [Baddeleyite, ZrO_2_]. In this study, a phosphate phase was not detected by XRD, similar to a study reported by Moinzadeh et al. [51].

Various types of CSC have similar chemical compositions and crystalline structures to white PCs, except in the presence of a radiopacifier.

Root canal cement should have sufﬁcient radiopacity to permit a clear differentiation between the cement and adjacent anatomical structures and to enable assessment of root canal filling quality, which can be assumed only through radiographic examination [52, 53]. The hydration process of MTA can be affected by the presence of bismuth oxide, because it is a part of the calcium silicate hydrate structure; in addition, it affects the precipitation of calcium hydroxide in the hydrated paste [54]. Zirconium oxide has a high atomic number and has been used as a radiopacifying material in glass ionomer cement. It has been used and selected as a radiopacifying material in certain types of CSC [55] and in dental and orthopedic applications as a biomaterial [56].

In a study performed by Silva et al. [57], where they compared CSCs containing bismuth oxide, with CSCs containing zirconium oxide (micro and nano), they found that CSCs containing zirconium oxide have higher compressive strength. Zirconium oxide combined with calcium silicate cement provides satisfactory physical and chemical properties and better biological reactions than bismuth oxide. Therefore, zirconium oxide can be used as a good substitute for a radiopacifier instead of bismuth oxide. Both types of ERRM (putty and paste) with EDX and XRD analysis showed the presence of Tantite (Ta_2_O_5_); this compound was not found in the remaining tested materials. This compound is a radiopacifier, and Ta_2_O_5_-containing cement has a strength comparable to that containing bismuth oxide [58]. However, in the present study, the strength of the materials was not in the parameter criteria of evaluation.

Furthermore, in this study, FTIR was used to identify the functional groups of the tested materials. FTIR is a helpful test in addition to XRD, as it permits the tracking of amorphous phases, particularly calcium silicate hydrate gel [41]. The important outcome in the FTIR results was the presence of a hydroxyl group. This provides a confirmation and good link with the XRD results, which indicated the presence of calcium hydroxide in all set forms of the tested materials. The FTIR results showed that the hydroxyl group was present significantly in all cured materials and in uncured white PC and both types of endosequence root repair materials (putty and paste). This is in agreement with a study performed by Han et al. [59], who studied the morphological and chemical analysis of MTA and found that the FTIR spectrum showed characteristic absorption bands of phosphate and hydroxyl groups. In addition, Camilleri et al. [41] reported that Biodentine and MTA Angelus contain calcium hydroxide in their composition.

In the results of this study, the authors found that calcium hydroxide was present in uncured materials, namely, white PC and both types of ERRM (putty and paste). This was assumed to be due to the high humidity in white PC because it is not sealed as other products that were used in this study and might be due to the manufacturing of ERRMs as premixed materials.

## 5. Conclusions

- SEM showed that the particle sizes and shapes differed between the materials tested in this study. White PC had the most irregular and large particle sizes. CSC had a smaller particle size and homogeneous shape, except for MTA Angelus.
- All CSCs were composed of crystalline structures which are the main structures of white PC. CSCs differed from PC by the presence of bismuth or zirconium.
- FTIR analysis showed that the different types of CSC and PC consist of hydroxyl groups, and it was most evident in white PC and both types of Endosequence root repair materials (both uncured and cured forms), which are assumed to be humidity issues and premixed products, respectively.

## 6. Clinical Relevance

The composition of CSC has a direct influence on the properties of these types of cement, which affects the clinical outcome of the treatment. Calcium hydroxide leached out of hydrated MTA is related to the release of calcium ions, and this process is affected by the presence of a radiopacifier that is included in the composition of the cement.

## Figures and Tables

**Table 1:** Materials used in the present study.

No.	Materials	Manufacturer
1.	White PC	Aalborg, Malaysia
2.	White ProRoot MTA	Dentsply Tulsa Dental, Tulsa, OK
3.	White MTA Angelus	Angelus, Brazil
4.	Biodentine	Septodont, UK
5.	Endosequence root repair material putty	Brasseler, Savannah, GA, USA
6.	Endosequence root repair material paste	Brasseler, Savannah, GA, USA

**Table 2:** The mean and standard deviation of the small and large particle sizes of different types of uncured CSC using the one-way ANOVA test.

	Mean and standard deviation of the small particle sizes (*μ*m)	One-way ANOVA test *P* value	Mean and standard deviation of the large particle sizes (*μ*m)	One-way ANOVA test *P* value
White PC	1.130 ± 0.221	0.000^*∗*^	42.822 ± 0.393	0.000^*∗*^
ProRoot MTA	1.132 ± 0.514		15.130 ± 1.566	
MTA Angelus	1.738 ± 0.528		51.822 ± 1.017	
Biodentine	0.972 ± 0.321		10.512 ± 0.816	
ERRM putty	0.194 ± 0.023		05.820 ± 0.905	
ERRM paste	0.194 ± 0.023		08.808 ± 0.788	

Values are expressed as means and standard deviations. *n* = 5. ^*∗*^*P* value less than 0.05 (*P* < 0.05) is considered a statistically significant difference.

**Table 3:** The comparison of the small particle size of the different types of uncured CSC using the Tukey post hoc test.

(I) tested materials	(J) tested materials	Mean difference (I-J)	*P* value
White PC	ProRoot MTA	−0.002	1.000
	MTA Angelus	−0.608	0.088
	Biodentine	0.158	0.976
	ERRM putty	0.936	0.003^*∗*^
	ERRM paste	0.936	0.003^*∗*^

^*∗*^The mean difference of less than 0.05 is significant.

**Table 4:** The comparison of the large particle size of the different types of uncured CSC using the Tukey post hoc test.

(I) tested materials	(J) tested materials	Mean difference (I-J)	*P* value
White PC	ProRoot MTA	27.692	0.000^*∗*^
	MTA Angelus	−9.000	0.000^*∗*^
	Biodentine	32.310	0.000^*∗*^
	ERRM putty	37.002	0.000^*∗*^
	ERRM paste	34.014	0.000^*∗*^

^*∗*^The mean difference of less than 0.05 is significant.

**Table 5:** The mean and standard deviation of the small and the large particle sizes of different types of cured CSC using the one-way ANOVA test.

	Mean and standard deviation of the small particle sizes (*μ*m)	One-way ANOVA test *P* value	Mean and standard deviation of the large particle sizes (*μ*m)	One-way ANOVA test *P* value
White PC	0.194 ± 0.078	0.000^*∗*^	25.410 ± 0.636	0.000^*∗*^
ProRoot MTA	0.970 ± 0.189		15.374 ± 1.555	
MTA Angelus	0.970 ± 0.092		28.020 ± 1.003	
Biodentine	1.130 ± 0.176		10.380 ± 0.303	
ERRM putty	0.194 ± 0.053		6.23 ± 1.748	
ERRM paste	0.194 ± 0.074		5.23 ± 0.932	

Values are expressed as means and standard deviations. *n* = 5. ^*∗*^*P* value less than 0.05 (*P* < 0.05) is considered a statistically significant difference.

**Table 6:** The comparison of the small particle size of the different types of cured CSC using the Tukey post hoc test.

(I) tested materials	(J) tested materials	Mean difference (I-J)	*P* value
White PC	ProRoot MTA	−0.77600^*∗*^	0.000^*∗*^
	MTA Angelus	−0.77600^*∗*^	0.000^*∗*^
	Biodentine	−0.93600^*∗*^	0.000^*∗*^
	ERRM putty	0.00000	1.000
	ERRM paste	0.00000	1.000

^*∗*^The mean difference of less than 0.05 is significant.

**Table 7:** The comparison of the large particle size of the different types of cured CSC using the Tukey post hoc test.

(I) tested materials	(J) tested materials	Mean difference (I-J)	*P* value
White PC	ProRoot MTA	10.03600	0.000^*∗*^
	MTA Angelus	−2.61000	0.016^*∗*^
	Biodentine	15.03000	0.000^*∗*^
	ERRM putty	19.18000	0.000^*∗*^
	ERRM paste	20.18000	0.000^*∗*^

^*∗*^The mean difference of less than 0.05 is significant.

**Table 8:** The main elements present in the different types of CSC (uncured and cured forms).

Elements material	Materials forms	C	O	Mg	Si	Ca	K	Al	Bi	Zr	Ta
White PC	Uncured	√	√	√	√	√	√	√			
	Cured	√	√	√	√	√	√	√			
White ProRoot MTA	Uncured	√	√	√	√	√		√	√		
	Cured	√	√	√	√	√		√	√		
White MTA Angelus	Uncured	√	√	√	√	√		√	√		
	Cured	√	√	√	√	√		√	√		
Biodentine	Uncured	√	√		√	√		√		√	
	Cured	√	√		√	√		√		√	
ERRM putty	Uncured	√	√		√	√				√	√
	Cured	√	√		√	√				√	√
ERRM paste	Uncured	√	√		√	√				√	√
	Cured	√	√		√	√				√	√

Carbon (C), oxygen (O), magnesium (Mg), silicon (Si), calcium (Ca), potassium (K), aluminum (Al), bismuth oxide (Bi), zirconium oxide (Zr), and tantalum oxide (Ta).

**Table 9:** The major compounds present in the different types of CSC (uncured and cured forms).

Elements materials	Materials form	(Ca_3_SiO_5_)	(Ca_2_SiO_4_)	(Ca_3_Al_2_O_6_)	(ZrO_2_)	(Bi_2_O_3_)	(Ta_2_O_5_)	(Ca (OH)_2_)
White PC	Uncured	√	√	√				√
	Cured	√	√	√				√
White ProRoot MTA	Uncured	√	√	√		√		
	Cured	√	√	√		√		√
White MTA Angelus	Uncured	√	√	√		√		
	Cured	√	√	√		√		√
Biodentine	Uncured	√	√	√	√			
	Cured	√	√	√	√			√
ERRM putty	Uncured	√	√	√	√		√	√
	Cured	√	√	√	√		√	√
ERRM paste	Uncured	√	√	√	√		√	√
	Cured	√	√	√	√		√	√

^*∗*^Tricalcium silicate (Ca_3_SiO_5_). ^*∗*^Dicalcium silicate (Ca_2_SiO_4_). ^*∗*^Tricalcium aluminate (Ca_3_Al_2_O_6_). ^*∗*^Zirconium oxide (ZrO_2_). ^*∗*^Bismuth oxide (Bi_2_O_3_). ^*∗*^ Tantalum oxide (Ta_2_O_5_). ^*∗*^Calcium hydroxide (Ca (OH)_2_).

**Table 10:** Absorption peaks of different types of uncured CSC using FTIR.

Materials	Wavenumber cm−1		
White PC	873.84	1139.46	3642.17
White pro-Root MTA	880.40	1155.64	—
White MTA Angelus	880.34	—	—
Biodentine	872.98	1413.19	—
ERRM putty	876.52	1064.98	3391.69
ERRM paste	880.17	1065.66	3391.67

**Table 11:** Absorption peaks of different types of cured CSC using FTIR.

Materials	Wavenumber cm^−1^		
White PC	872.97	1108.39	3640.37
White proRoot MTA	876.59	1112.40	3640.96
White MTA Angelus	880.48	1390.92	3638.37
Biodentine	872.87	1434.27	3637.74
ERRM putty	852.71	1426.66	3644.30
ERRM paste	849.35	1061.87	3644.33

## Data Availability

The data used to support the findings of this study are available from the corresponding author upon request.
